# Dance Fitness Action Recognition Method Based on Contour Image Spatial Frequency Domain Features and Few-Shot Learning

**DOI:** 10.1155/2022/1559099

**Published:** 2022-06-08

**Authors:** Qinglong Chi, Lifeng Li

**Affiliations:** ^1^Dance Teaching and Research Office, Shandong Sport University, Jinan 250000, China; ^2^Music Teaching and Research Group, Xiamen Foreign Language School, Xiamen 361000, China

## Abstract

In recent years, the research work of artificial intelligence technology has progressed rapidly, and various classic Few-Shot learning models have achieved unprecedented success in many artificial intelligence application fields. These include face recognition, object classification detection and tracking, speech recognition, and natural language processing, which greatly facilitate our lives. This paper aims to identify dance fitness movements based on contour image spatial frequency domain features and Few-Shot learning technology. This paper proposes a Few-Shot learning method based on contrastive average loss for Few-Shot learning. This method makes the learned model more representative by improving the loss function and performing a normalization process, and it proposes a feature extraction algorithm that combines improved LBP and HOG for action recognition technology. The experimental results show that the recognition accuracy of the algorithm in this paper is 93.10%, 90.30%, and 92.70% for walking, opening hands, and running, respectively. This illustrates the effectiveness of the fusion feature algorithm.

## 1. Introduction

In the early days, research based on images and videos was an important part of human action recognition methods. With the continuous development of computer technology, intelligent machines and equipment have gradually attracted widespread attention from human beings. These methods mainly take the RGB information collected by the camera equipment as the research object and use the features such as texture and color information in the two-dimensional image to perform action recognition. However, the changes of lighting conditions, background interference, and occlusion and self-occlusion problems cannot be solved well, which has become the bottleneck of this type of research method. In order to enable deep learning models to overcome the problem of relying on large-scale data and obtain the ability to quickly learn new categories of things similar to humans, Few-Shot learning came into being.

The general action recognition method works by extracting features from the original input and then training a classifier using those features. Robust feature representations must be obtained to ensure the accuracy of the final algorithm, which necessitates a significant amount of computational and testing effort. Massive labeled data are usually difficult to obtain in real-world applications. On the one hand, some industries find it difficult to collect image data for a variety of reasons, including privacy and security; on the other hand, even if image data are collected, labelling it is often costly. These disadvantages severely limit the application of image classification models. As a result, for dance fitness action recognition, it is necessary to investigate the spatial frequency domain features of contour images as well as Few-Shot learning technology.

On the basis of predecessors, this paper has the following two innovations for human body recognition research: (1) a contrastive average loss-based Few-Shot learning method is proposed. Few-Shot learning is a commonly used method in human action recognition research, but the traditional Few-Shot learning method will be hampered by the action's noise and background processing. This problem can be solved using the Few-Shot learning method based on contrastive average loss proposed in this paper. (2) The three movements of running, walking, and opening hands are used to research dance fitness movement recognition. Because these three movements are the most common in dance and fitness, and because other movements are based on their extensions, research on these three movements can better represent the entire movement.

## 2. Related Work

There are numerous studies on human action recognition that are related. To compensate for the flaws of traditional algorithms, Tarn C et al. used the Few-Shot learning method to fit data online. Their proposed method was tested on ten datasets using different collision energy settings and instruments such as Velos, QE, Lumos, and Sciex. Their findings show that Few-Shot learning can improve prediction accuracy while using virtually no computational resources [1]. Research performed by Szcs and Németh aims to aid medical workers' work by analyzing pathological X-ray recordings with only a few images. They propose the Deep Neural Network-based Dual View Matching Network (DVMN), an improved method that addresses the problem of Few-Shot learning and different views of pathological records in images. Its main contribution is the use of convolutional neural networks in image representation for feature extraction and multiview processing [2]. Li et al. proposed Joint Distance Maps, a simple and effective method for encoding the spatiotemporal information of skeleton sequences into color texture images, based on the good performance of deep learning (JDMs). They also used convolutional neural networks to extract distinguishing features from JDMs for recognizing human action and interaction. The proposed method has been validated by state-of-the-art results in single-view and cross-view settings on the large RGB + D dataset and the small UTD-MHAD dataset [3]. SkeletonNet, a deep learning framework for skeleton-based 3D action recognition, was investigated by Ke et al. They begin by extracting features based on body parts from each frame of the skeleton sequence. When compared to the original coordinates of the skeletal joints, the proposed features are translation, rotation, and scale invariant [4]. Wang et al. analyzed human activity recognition from videos using multimedia and convolutional neural network features. They feed frame-level CNN sequence features to a long short-term memory (LSTM) model for video activity recognition, treating video as a sequence of frames [5]. To process video data, Ullah et al. proposed an action recognition method using convolutional neural networks and deep bidirectional LSTM-LSTM networks. First, every six frames of video are analyzed for deep features, which helps to reduce redundancy and complexity. Then, using a DB-LSTM network, order information between frame features is learned, with multiple layers stacked together in the forward and reverse passes to increase the depth of the network. By analyzing features within specific time intervals, the method can learn long-term sequences and process lengthy videos. On three benchmark datasets, UCF-101, YouTube 11 Actions, and HMDB51 [6], their experimental results show that the proposed method achieves significant improvement in action recognition compared to existing action recognition methods.

Many studies on human actions are based on machine learning methods, and the extraction of video information is emphasized, as evidenced by a review of related research. These kinds of processing methods are quite complicated.

## 3. Few-Shot Learning Based on Contrastive Average Loss

### 3.1. Introduction to Few-Shot Learning

**Figure 1:**
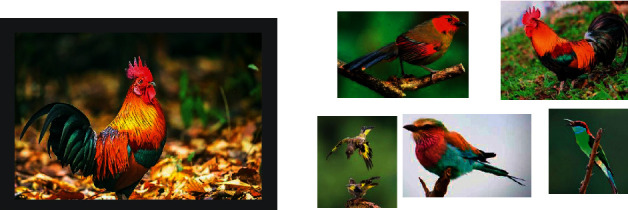
Example of rooster single-sample image classification.

In recent years, Few-Shot learning is a subfield of machine learning [7, 8]. The definition of machine learning is that a computer program learns from experience (experiences, E) to solve a certain task (task, T) and perform a certain performance measure (performance, P). Performance P on task T improves with experience E. While Few-Shot learning is a class of machine learning problems (specified by E, T, and P), where E contains little supervision information for the target T. Specific to the image classification [9, 10] task based on Few-Shot learning, T is the image classification task, E is a small number of labeled pictures and prior knowledge used for the classification task, and the performance measure P is the accuracy rate. According to the definition of machine learning, the classification task should be to improve the experience E based on the measured accuracy, while the few labeled images are fixed. Therefore, only the part corresponding to the prior knowledge can be improved. Specifically, the prior knowledge may be some original images of other categories or a pretrained model.  Figure 1 is an example of rooster single-sample recognition. The model only needs to judge which birds on the right are roosters based on the rooster image on the left.

**Figure 2:**
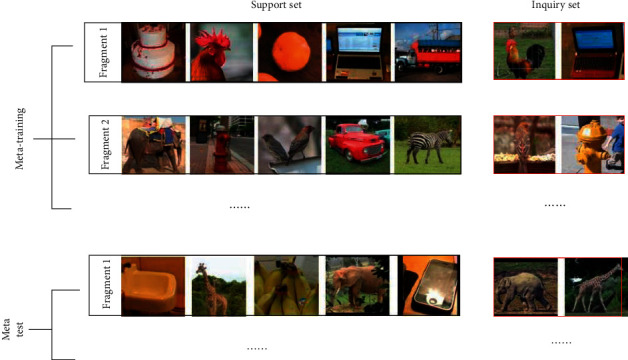
Schematic diagram of segment training mechanism for c-way k-shot image classification, where *c* = 5 and *k* = 1.

Therefore, the training method of Few-Shot learning is different from the training method of conventional deep learning models [11–13]. Specific to the image classification problem, the training process usually contains two datasets, namely the support dataset and the query dataset. Formally, the training set learned by Few-Shot contains multiple samples of different classes. The model is required to learn how to distinguish the c classes from the Few-Shot with the number of training sets *ck*. Such a task is called a c-way k-shot classification task. The value of *k* is usually 1 or 5. When *k* is 1, this Few-Shot learning is called one-shot learning, as shown in Figure 2:

The core problem of Few-Shot learning is insufficient sample size. In order to “learn” from a small number of samples to a deep learning model that can be generalized, the current main solutions can be divided into three categories: Few-Shot learning methods based on data augmentation, metric learning, and meta-learning. As stated in Chapter 1, the current research status at home and abroad, the probability-based generative model in the nondeep learning stage is the mainstream of the Few-Shot classification task; and the current mainstream is the deep learning-based discriminative model. So far, there are three main methods for using discriminative models to solve the Few-Shot classification problem: data augmentation, metric learning, and meta-learning. They solve the problem of Few-Shot image classification from three aspects: directly expanding the diversity of label samples, using prior knowledge (training set data) to learn better feature models, and optimizing hyperparameters to make the model learn quickly. The following three methods are briefly introduced.

#### 3.1.1. Few-Shot Learning Based on Data Augmentation

The data augmentation method based on Few-Shot learning is a method to enhance data and improve data diversity through prior knowledge. There are two types of data augmentation methods, one is a generative model that generates new samples through transformation; the other is a transduction model that uses the model to label unlabeled samples and then further trains the model. The specific working method is shown in Table 1. The converter inputs the unlabeled image *u* and the reference sample image *x*, and its label *y* and outputs the synthesized image *x* and label *y*, which are used to enhance the training dataset of Few-Shot.

Image processing [14, 15], zoom-in and zoom-out, random cropping, adding noise, mirror flipping, and other basic data augmentation methods based on generative models are among the most basic. These manual transformers can create new images that have the same tags as the old ones. The amount of data generated above for Few-Shot learning is far insufficient to improve the model's generalization ability. Many new methods have been developed as a result of the development of deep learning, the majority of which are based on autoencoders (AE) or adversarial generative networks (GAN) [16]. DAGAN learns on the training set using a generator that combines U-Net and ResNet, as well as a discriminator that uses DenseNet. This trained GAN model is then used to generate new sample images for Few-Shot image classification on the test set [17, 18].

**Figure 3:**
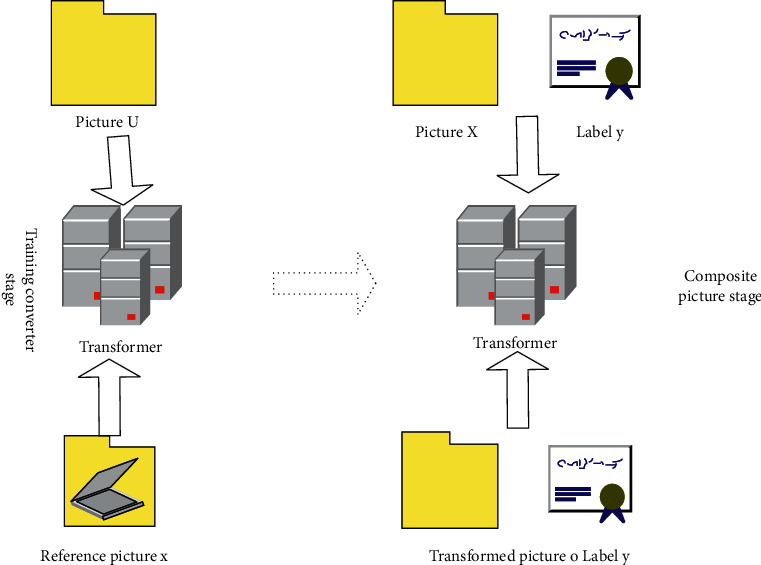
Schematic diagram of data synthesis method based on generative model.

It can be seen that the difference between this type of data expansion method and other Few-Shot learning methods is that there are two more steps before training image classification, as shown in Figure 3, which are training converters (generators), and then synthesizing new data.

#### 3.1.2. Metric-Based Few-Shot Learning

Metric learning refers to extracting features from samples and embedding them into a feature space with a small dimension, and then using the distance between two features to measure the similarity of two samples. Commonly used distances in feature space include Euclidean Metric and Cosine similarity [19, 20].

**Figure 4:**
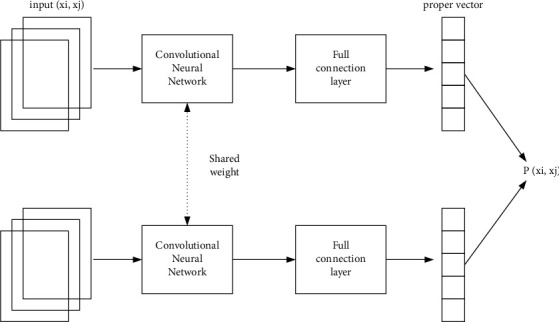
Siamese network structure diagram.

*(1) Siamese Network*. Siamese network is the first to introduce metric learning into Few-Shot learning. As shown in Figure 4, the network structure consists of two convolutional neural networks (CNNs), which input a pair of samples (*x*_*i*_, *y*_*i*_). At the same time, the two CNNs share the convolution kernel weight, then output two feature vectors through the fully connected layer, and finally output the similarity score *p*(*x*_*i*_, *y*_*i*_) of the two samples according to the distance between the two vectors. The distance metric function of the two features is directly a weighted 1 function, and the loss function of the network structure is shown in the following equation:

(1) $$\begin{array}{c} \nabla =\sum_{q}y_{ij}\log\;p\left(x_{i},y_{i}\right)+\left(1-y_{ij}\right)\log\left(1-p\left(x_{i},y_{i}\right)\right). \end{array}$$

Among them, *y*_*ij*_ = 1 when *x*_*i*_ and *x*_*j*_ belong to the same category. After the model is trained, the parameters of the Siamese network are fixed and tested on the Few-Shot task, and then, the nearest neighbor classifier (NearestNeighborClassifier) is used for classification.

*(2) Matching Network*. Unlike Siamese networks, which share parameters for training and test sets, matching networks are the first to design a piecemeal training mechanism. The matching network adds a support set attention model. Specifically, in the training process, the convolutional neural network is used to extract features for the support set and the query set, and then, the attention weight corresponding to each support set is calculated according to the sample features of the query set, and then calculate the possible labels of the query set samples according to the attention weights, and finally use the cosine similarity to measure the matching degree of the support set samples and the query set samples. The calculation formula of attention is shown in the following equation:

(2) $$\begin{array}{c} \alpha \left(x,x_{i}\right)\frac{\exp\left[c\left(f\left(x\right),g\left(x_{i}\right)\right)\right]}{\sum_{i=1}^{k}\exp\left[c\left(f\left(x\right),g\left(x_{i}\right)\right)\right]}, \end{array}$$

where *x* represents the query set sample, *x*_*i*_ represents the *i*-th support set sample, *c*(,) represents the cosine distance, and *f*() and *g*() represent the feature extraction function of the query set and support set, respectively.

*(3) Prototype Network*. Since the matching network studies the single-sample problem, the number of each sample learned by Few-Shot is less than 20. In order to solve the problem of solving the similarity of feature space in the case of multiple samples of each type, the prototype network has designed the concept of prototype. First, the support set samples are embedded in the feature space, and then, the center of each class of the support set is designed as a prototype. The prototype calculation formula is shown in the following equation:

(3) $$\begin{array}{c} C_{k}=\frac{1}{S_{k}\left(x_{i},y_{i}\right)}\sum_{\left(x_{i},y_{i}\right)\in S_{k}}f_{\varphi }\left(x_{i}\right). \end{array}$$

Among them, *f*_*φ*_(*x*_*i*_) represents the feature extraction function, and *S*_*k*_ represents the support set containing *k* samples for each class.

Then, the similarity is calculated by calculating the Euclidean distance *d*(,) between the query set and the prototype. In the test, the normalized exponential function is used to calculate the distance between the test sample and the prototype of the support set. The specific formula is shown in the following equation.

(4) $$\begin{array}{c} p_{\varphi }\left(y=k|x\right)=\frac{\exp\left[-d\left(f_{\varphi }\left(x_{i}\right),c_{k}\right)\right]}{\sum_{k}\exp\left[-d\left(f_{\varphi }\left(x_{i}\right),c_{k}\right)\right]}. \end{array}$$

Prototype networks do not suffer from class imbalance since there is only one prototype per class. Also since the prototype can only capture the mean of the support set, naturally its variance information will be lost. The features extracted from the support set are updated by extracting the features from the query set of the task as environmental features, so that each task has its own different feature output, so as to solve the problem that the variance information will be lost.

**Figure 5:**
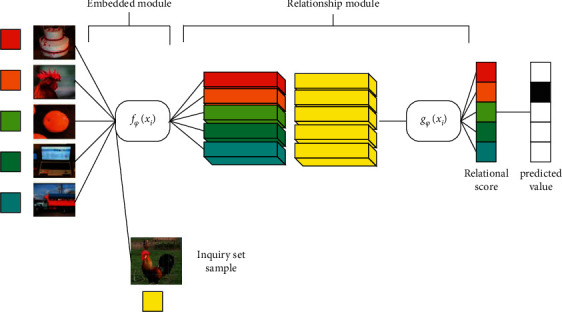
Schematic diagram of the relationship network structure.

*(4) Relationship Network*. As shown in Figure 5, the relational network model mainly includes two modules: an embedded module and a relational module. The former is the feature extraction module that every metric learning must have; and the relational module is the metric module, which directly connects the sample features of the query set and the support set, and then input into a convolutional neural network and directly classify and score through the fully connected layer. The classification scoring formula is shown in the following equation:

(5) $$\begin{array}{c} r_{i,j}=g_{\phi }\left[C\left(f_{\phi }\left(x_{i}\right),f_{\phi }\left(x_{j}\right)\right)\right],\;i=1,2,\ldots ,C. \end{array}$$

Among them, *x*_*i*_ and *x*_*j*_ represent the support set and query set samples, respectively; *f*_*φ*_(*x*_*i*_) represents the embedding function; *c*(,) represents the concatenation of the embedded feature maps; and *g*_*ϕ*_(*x*_*i*_) represents the relation function.

#### 3.1.3. Few-Shot Learning Based on Meta-Learning

Metric learning, hyper-parameter optimization-based meta-learning, and model-based meta-learning are some of the meta-learning-based methods for Few-Shot image classification. Metric learning, and thus its widespread use, has become a separate branch among them. Model-based meta-learning methods are frequently combined with metric learning and hyperparameter optimization meta-learning. As a result, the following sections focus on hyperparameter optimization-based meta-learning methods.

The core idea of MAML (Model-Agnostic Meta-Learning) model is to set initialization parameters suitable for fast learning for basic learning model parameters through cross-task training strategy. In this way, the base learning model can generalize well to new tasks using only a few samples of the support set. Specifically, the parameter update formula for the basic learner of task *T* is shown in the following equation:

(6) $$\begin{array}{c} \theta_{b}^{T}=\theta_{b}-\alpha \nabla_{\theta_{b}}L\left(D^{T},\theta_{b}\right), \end{array}$$

where *α* is the learning rate, *L*(*D*^*T*^, *θ*_*b*_) is the loss function of task *T* on the support set, and *θ*_*b*_ is the initialization parameter of the basic learner.

In the meta-learning stage, MAML optimizes the meta-learner model by balancing the loss corresponding to parameter *θ*_*b*_^*T*^ of the basic learning model on multiple tasks. The specific formula is shown in the following equation:

(7) $$\begin{array}{c} \theta_{m}=\theta_{m}-\beta \nabla_{\theta_{m}}L\left(D^{T},\theta_{b}^{T}\right). \end{array}$$

It is worth noting that the meta-learning model and the basic learning model in MAML in Table 2 are the same model, that is, parameter *θ*_*m*_=*θ*_*b*_. In fact, there are many MAML-based variant models. The MAML-based variant models and their improvement points in recent years are shown in Table 2.

### 3.2. Few-Shot Learning Based on Contrastive Average Loss

In metric learning, a prototype network is a representative algorithm framework. It uses the feature embedding module to process the data and represent it as a prototype for a specific category. The metric module is a parameter-free module that compares query set samples to all prototypes and chooses the prototype category with the shortest distance as its own category. When the algorithm creates category prototypes, however, it only takes into account the relationship between samples and similar prototypes, which can cause issues in more complex situations.

This paper proposes a new loss function based on the prototype network. In comparison with the average loss (CDM), the loss function compares the difference between the sample and different prototypes at the same time, reducing the difference between Few-Shot and the same type of prototype while increasing the differences between samples and different types of samples. This will improve the feature embedding function's ability to represent features, making the learned prototypes more representative. Prototypes of various categories are more differentiated, which improves the model's generalization ability. The method is tested on a benchmark dataset for image classification, and the results show that it can effectively improve the model's performance.

The loss function proposed in this paper will consider the relationship between samples and prototypes of different categories at the same time. Through the adjustment of hyperparameters, it can flexibly convert between the overall category and some categories. Experiments show that this method can improve the performance of the model on the dataset. Note the training dataset *D*={(*x*_*i*_, *y*_*i*_)_*i*=1_^*N*^}. For a support set sample, the number of categories is *k*, and the prototype of a category is denoted as *C*_*k*_. The class prototype to which sample *x*_*i*_ belongs is *C*_*i*_, and the distance between sample *x*_*i*_ and class prototype *C*_*j*_ is denoted by *d*. The measurement method of distance can be selected by yourself. The distance contrast is recorded as *l*, and the optimization goal is to minimize it, as shown in the following equation:

(8) $$\begin{array}{c} l=d^{2}\left(x_{i},c_{i}\right)-\frac{1}{m}\sum_{j\neq i}d^{2}\left(x_{i},c_{i}\right). \end{array}$$

In the formula, *m* is an adjustable hyperparameter. Its function is to adjust the number of comparisons between a single sample and different types of prototypes, and its maximum value is *k* − 1. In machine learning, gradient descent is a commonly used optimization algorithm. According to the amount of data used to update the parameters each time, it is divided into batch gradient descent, mini-batch gradient descent, and stochastic gradient descent (SGD). After the model training is completed, given a query set sample, the corresponding prediction is as shown in the following equation:

(9) $$\begin{array}{c} y_{q}=\underset{k}{\arg\min}d\left(f_{\theta }\left(x_{q},c_{k}\right)\right). \end{array}$$

That is, the sample will be marked as the class of the closest prototype to it. The distance metric part of this loss function consists of two parts. Considering that the proportion of the difference between the two may have a certain impact on the results, parameters can be added before the two parts to control, such as the following formula:

(10) $$\begin{array}{c} l=\alpha \times d^{2}\left(x_{i},c_{i}\right)-\left(1-\alpha \right)\frac{1}{m}\sum_{j\neq i}d^{2}\left(x_{i},c_{i}\right). \end{array}$$

The value range of *α* is between 0 and 1. By adjusting the value, the numerical proportion of different parts in the loss can be adjusted. However, after experiments, the effect of *α* is very limited, and the addition or not has little effect on the experimental results. And *α* is a hyperparameter, and the adjustment of the parameters must be considered after adding it. Taking it into consideration, it is more secure to remove it; that is, the part of the lost contrast remains unchanged.

## 4. Contour Image Spatial Frequency Domain Feature Extraction Algorithm

### 4.1. HOG Feature Extraction Algorithm

The essence of HOG is to rely on the statistics of gradients in the image. This gradient information is usually at the edge of the image, and the directional density distribution at the edge can better reflect the information and shape of the local target in the image. The primary basis of HOG is to segment the image and divide it into connected regions called cell units; next, we need to collect the edge or gradient direction histogram of each pixel in the segmented cell unit above, and finally combine them to obtain the feature descriptor we need. These combined directional gradient histograms need to be diffused from the local area, and the contrast normalization operation is performed in a larger area in the image. This larger area is called a block, which is an interval. The main operation steps are to first calculate the density of the histogram of each direction in the whole area, and then normalize the whole cell unit according to the calculated density, so as to reduce the influence of light and shadow on the detection result.

The concept of directional gradient histogram (HOG) was proposed earlier, and it has many advantages compared with other algorithms. First, it is not very sensitive to the effects of optical and geometric distortions in the image, because all its operations are performed in local grid cells. The second point is that under the condition that the local optical normalization is relatively high, the sampling in the spatial domain is relatively coarse, and the sampling in the direction is relatively fine, and some relatively small movements of the human body can be allowed. Usually, these relatively small actions are ignored and have little effect on the final result. Through the above content, we can find that the directional gradient histogram will have a wide range of applications in human body recognition in images.

#### 4.1.1. Calculate the Image Gradient

The gradients of the horizontal and vertical coordinates in the image are calculated, respectively, to obtain the value of the gradient direction of each pixel in the image. Through this derivation method, we can reduce the sensitivity to light and obtain information such as human contours and textures in the image.

The gradient component of the pixel point (*x*, *y*) is shown in following equations:

(11) $$\begin{array}{c} G_{x}\left(x,y\right)=H\left(x+1,y\right)-H\left(x-1,y\right), \end{array}$$

(12) $$\begin{array}{c} G_{y}\left(x,y\right)=H\left(x,y+1\right)-H\left(x,y-1\right). \end{array}$$

The gradient magnitude and direction of the (*x*, *y*) point are as shown in following equations:

(13) $$\begin{array}{c} G\left(x,y\right)=\sqrt{G_{x}{\left(x,y\right)}^{2}+G_{y}{\left(x,y\right)}^{2}}, \end{array}$$

(14) $$\begin{array}{c} \alpha \left(x,y\right)=\;\tan^{-1}\left(\frac{G_{y}\left(x,y\right)}{G_{x}\left(x,y\right)}\right). \end{array}$$

In the directional gradient histogram, the following methods are often used: in the first step, the gradient operator is used to perform convolution calculation to obtain the gradient component in the horizontal direction; the second step is to use the gradient operator to perform convolution calculation to obtain the gradient component in the vertical direction; and finally, formula (13) and (14) are used to calculate the gradient direction and size at the pixel position.

#### 4.1.2. Construct the Gradient Direction Histogram for Each Unit

**Figure 6:**
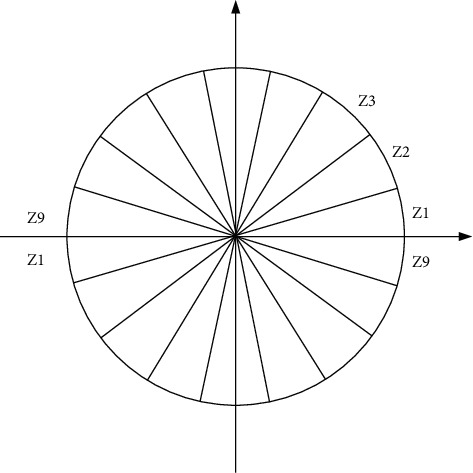
Schematic diagram of gradient direction segmentation.

This step is mainly to provide a code to the local area, but without destroying the weak sensitivity to human targets while encoding. First, the processed image is divided into several units called cells. Here, we assume that a cell contains 36 pixels, that is, 6 × 6, and the gradient information of these 36 pixels is counted through the histogram. A cell has a gradient direction of 360 degrees, and we divide it into 9 parts, as shown in Figure 6.

### 4.2. Classifier

Now add the following explanation: SVM is an effective method to solve the problem of Few-Shot pattern recognition. Finding the generalized optimal classification hyperplane is in high-dimensional space. In order to realize the recognition of multiple categories, it is necessary to make corresponding improvements to the SVM.

At present, many researchers have extended SVM and proposed many excellent algorithms to solve multiclass classification problems. For example, using multiple two-class classifiers and other methods in action recognition, the commonly used kernel functions of SVM include linear kernel and histogram. In this paper, the two factors of SVM classification accuracy and computational complexity are comprehensively considered, and through experimental comparison, the histogram cross-kernel is used as the kernel function of the classifier. Its expression is as in the following equation:

(15) $$\begin{array}{c} K\left(X_{i},X_{j}\right)=\sum_{n=1}^{m}\min\left(a_{n},b_{n}\right). \end{array}$$

Among them, *X*_*i*_, *X*_*j*_ is two arbitrary eigenvectors, *a*_*n*_, *b*_*n*_ is the eigenvalue of the nth dimension, and *m* is the dimension of the eigenvector. Compared with the other two kernel functions, the histogram cross-kernel has the characteristics of low computational complexity and good classification effect. Then, we input the previously obtained feature vector based on the histogram cross-kernel SVM to obtain the final classifier. After obtaining the test set features, we use the classifier just obtained to make corresponding predictions on the results, and finally achieve action recognition.

### 4.3. Improved LBP Algorithm

When the improved LBP encodes, firstly, the difference between the gray value of the pixel at the center position and the gray value of its adjacent pixels is taken separately, and then, the difference is added and the average value is obtained to obtain the threshold M, as shown in the following equation:

(16) $$\begin{array}{c} M=\frac{1}{p}\sum_{i=0}^{p-1}\left|g_{i}-g_{c}\right|. \end{array}$$

Then, we use the gray value of the neighboring pixels and the gray value of the center pixel to perform a subtraction operation in a certain order (clockwise or counterclockwise) to get the absolute value, and then add it compare with the threshold *M*. If the value is greater than *M*, it is coded as 1; otherwise, it is coded as 0. Its coding formula is shown in the following equation:

(17) $$\begin{array}{c} S\left(g_{p}-g_{c}\right)=\left\{\begin{array}{cc} 1, & \left|g_{p}-g_{c}\right|>M,\\ 0, & \left|g_{p}-g_{c}\right|\leq M. \end{array}\right. \end{array}$$

Finally, we convert the encoding. Since the computer encoding system uses binary data, we convert it into decimal data that are easy to calculate, as shown in the following equation:

(18) $$\begin{array}{c} {\text{LBP}}_{P.R}^{*}=\sum_{i=1}^{p-1}S\left(g_{p}-g_{c}\right)2^{p}. \end{array}$$

### 4.4. Feature Fusion

Multifeature fusion can retain the effective identification information of various features and can also remove the redundant and invalid parts of various features to a certain extent. Many current feature fusion methods directly merge two sets of feature vectors into a new feature vector in a serial manner. In order to solve this problem, this paper compresses the two features extracted at the beginning and performs weighted fusion, so as to achieve effective fusion of feature vectors, and at the same time appropriately reduce the dimension of feature vectors.

At present, there have been many related researches on multifeature fusion, including the fusion of HOG features and SIFT features, the fusion of HOG features and ordinary LBP features, and the fusion of SIFT features and LBP features. The SIFT feature is a scale-invariant feature transform. SIFT features remain invariant to rotation, scale scaling, brightness changes, etc., and maintain a certain degree of stability for viewing angle changes, affine transformations, occlusions, and noise, and are relatively stable local features. However, it detects too few feature points for blurred images and images with smooth edges, resulting in a low recognition accuracy. In the follow-up experiments, the proposed algorithm is also compared with these existing fusion methods.

**Figure 7:**
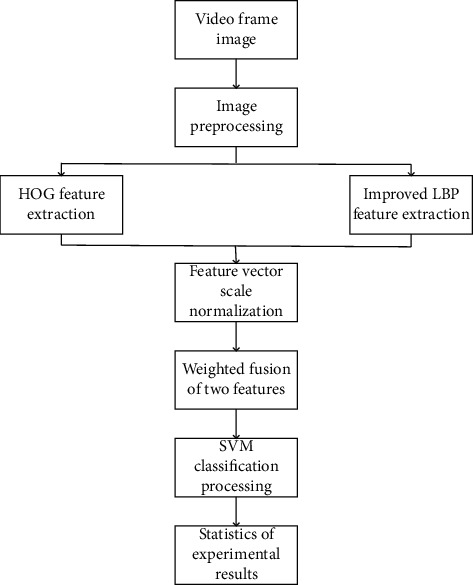
Flowchart of feature fusion algorithm.

Due to the above shortcomings of a single feature, this paper proposes a method based on the fusion of HOG features and improved LBP features. Feature fusion first needs to extract a large amount of feature information and then compares the information. Although this can enhance real-time performance, it will lead to missing information and inaccurate results. However, if we adopt serial fusion, although the recognition accuracy can be improved, it will lead to an increase in the amount of data and reduce the detection efficiency. Select a video from Weizmann and YouTube, respectively, and then extract the video frame image, and perform grayscale and normalization processing on the image. It is mainly to standardize Gamma and color space and also to reduce the influence of lighting and other factors on the detection results. Therefore, this paper adopts the method of weighted fusion of HOG features and improved LBP features. The algorithm flowchart is shown in Figure 7.

(1) In an image, the exposure of the local surface layer accounts for a large proportion of the texture intensity. By normalizing the image, we can greatly reduce the lighting and the effect of shadows on the local part of the image. Typically, we first convert the image to grayscale. The Gamma compression formula is shown in equation (19), and Gamma is represented by 1. In this experiment, the value of 1 is taken as 0.5.
  (19) $$\begin{array}{c} I\left(x,y\right)=I{\left(x,y\right)}^{\gamma }. \end{array}$$
(2) Normalize the scale of the two feature sets obtained.
(3) Use a weighted method to perform feature fusion to obtain the final feature set, as shown in equation (20). Among them, *α*, *β* ≥ 0 is the weight, which satisfies *α*+*β*=1.
  (20) $$\begin{array}{c} T\left(l\right)=\alpha L\left(I\right)+\beta H\left(I\right). \end{array}$$
(4) Use the SVM classifier to identify in which the SVM kernel function adopts the histogram cross-kernel.

## 5. Recognition and Analysis of Dance Fitness Movements

**Figure 8:**
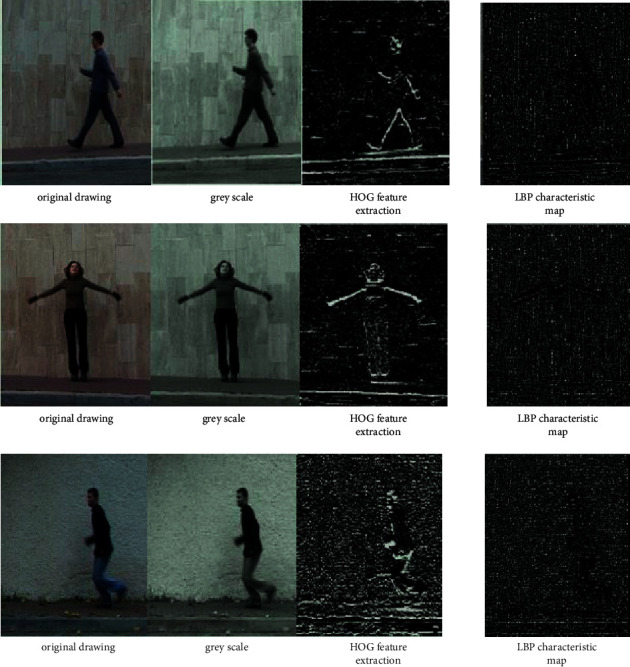
HOG and LBP feature maps under different actions.

Dance fitness movements are complex, especially under high-speed movement, various movements are difficult to capture and cannot achieve good results. In this paper, three common movements in dance fitness are selected for identification and analysis. The three movements are walking, opening hands, and running. The feature extraction structure of a single frame is shown in Figure 8.

As shown in Figure 8, in different actions, the contours extracted from the two feature maps are relatively clear, but some details are still blurred. Therefore, the action recognition for dance fitness still needs to be optimized.

For the Omniglot dataset, the training epoch = 100, the learning rate is 0.001, and the dynamic adjustment strategy is adopted, and the learning rate is adjusted to one-half of the original every 20 rounds. The value of episode is set to 100 in one epoch, and the number of categories in each episode is set to 60. By adjusting the number of samples for each category in the support set to 1 and 5, and the number of categories to 5 and 20, the corresponding n-way k-shot training is completed. For the miniImageNet dataset, due to the large amount of data, the total number of training rounds is set to 200, that is, epoch = 200, and the learning rate is 0.001. The dynamic adjustment strategy of the learning rate is the same as above, and the value of episode in each epoch is also 100. When the image is input, it will be scaled to a size of 84 ∗ 84. In order to speed up the convergence, the RGB channels of the image will be normalized. During SeProtNet training, the value of *γ* is set to 16.

**Figure 9:**
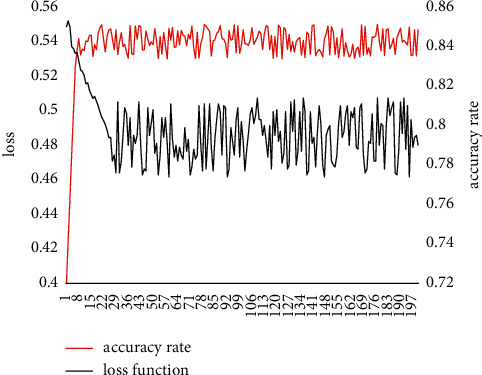
Iterative curve of accuracy loss in 5-way 5-shot.

The training process under the 5-way 5-shot setting is shown in Figure 9. When the number of iterations is 161,000, the image classification model based on Transformer Few-Shot learning achieves the highest accuracy rate of 85.28% on the validation set. It can be seen from Figure 9 that the image classification model based on Transformer Few-Shot learning in the case of 5-shot, when the accuracy rate and loss basically converge, the fluctuation range is much smaller than the 1-shot setting.

As shown in Table 3, in the testing phase, the trained model performance is measured by randomly building 600 segment tasks. The accuracy of the Transformer Few-Shot-based learning model is better than the other methods in both settings, with 66.75% accuracy under 1-shot setting and 82.05% under 5-shot setting. Among them, the prototype network, that is, the model without Transformer and group regularization, is the basic network of this model. Compared with the prototype network, the accuracy of this model has been greatly improved, with an increase of 6.43% under the 1-shot setting and 4.03% under the 5-shot setting.

**Figure 10:**
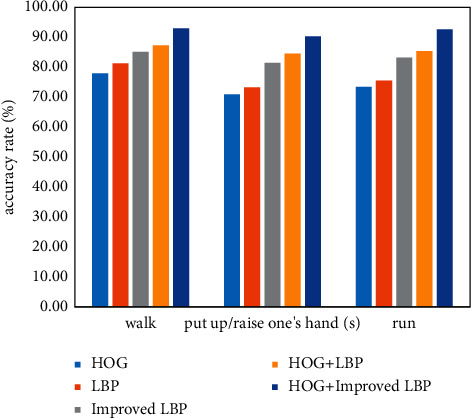
Recognition accuracy of different feature extraction algorithms.

As shown in Figure 10, the three actions of walking, opening hands, and running are analyzed under the dataset. The accuracy of different algorithms is different, but the recognition efficiency of the feature extraction algorithm that combines HOG and improved LBP algorithm has always been high. The recognition accuracy rates of walking, opening hands, and running are 93.10%, 90.30%, and 92.70%, respectively. This illustrates the effectiveness of the fusion feature algorithm.

## 6. Conclusions

Few-Shot and data imbalance are frequently symbiotic in practical applications. Data augmentation, knowledge transfer between similar categories, data synthesis, structured knowledge graph, domain adaptive learning, and model solvability are all strategies that can help Few-Shot learn better. At the moment, Few-research Shot's is primarily focused on image data modeling and processing. Many fields of supervised learning, such as video object detection and semantic segmentation, have an urgent need for Few-Shot learning, and other directions are also worth exploring. Furthermore, in the future, directions such as natural language processing and recommendation systems will be important application scenarios for Few-Shot learning.

## Figures and Tables

**Table 1:** Few-Shot image classification method based on data augmentation.

Type	Input (*u*, *x*, *y*)	Converter *t*	Output (*x*, *y*)
Generating model	Original (*u*_*i*_, *x*_*i*_, *y*_*i*_)	Transformation model based on training set learning	(*t*(*u*_*i*_), *y*_*i*_)

Transduction model	Unmarked picture	Classifier based on training set learning	(*x*_*i*_, *y*_*i*_)

**Table 2:** Improved model based on MAML framework.

way	Improvement point
MAML	

BMAML	A parameter-free reasoning model based on MAML is designed to solve the uncertainty problem of MAML model.

MT-net	Simplify the parameter space of MAML meta-learning model into a subspace composed of each layer of activation space, thus reducing the difficulty of fast learning.

TAML	Unbiased task unknowable experience is added to the initial model to solve the problem of poor performance of MAML framework in meta-training.

LEO	Combining MAML with metric learning, the model can quickly learn hyperparameters in low-latitude embedded space.

**Table 3:** Few-Shot classification results based on different methods.

way	1-Shot (%)	5-Shot (%)
HOG	60.37	78.02
LBP	62.08	78.63
Improved LBP	62.64	80.51
HOG + LBP	64.60	79.51
HOG + Improved LBP	66.87	82.44

## Data Availability

The data used to support the findings of this study are available from the corresponding author upon request.
